# Temporal dynamics of the gut microbiota in people sharing a confined environment, a 520-day ground-based space simulation, MARS500

**DOI:** 10.1186/s40168-017-0256-8

**Published:** 2017-03-24

**Authors:** Silvia Turroni, Simone Rampelli, Elena Biagi, Clarissa Consolandi, Marco Severgnini, Clelia Peano, Sara Quercia, Matteo Soverini, Franck G. Carbonero, Giovanna Bianconi, Petra Rettberg, Francesco Canganella, Patrizia Brigidi, Marco Candela

**Affiliations:** 1 Unit of Microbial Ecology of Health, Department of Pharmacy and Biotechnology, University of Bologna, Bologna, 40126 Italy; 2 Institute of Biomedical Technologies – National Research Council (ITB-CNR), Segrate, Milan 20090 Italy; 3 Department of Food Science, University of Arkansas, Fayetteville, AR 72704 USA; 4 Department for Innovation in Biological, Agrofood, and Forest Systems, University of Tuscia, Viterbo, 01100 Italy; 5 Radiation Biology Department, Institute of Aerospace Medicine, German Aerospace Center (DLR), Cologne, 51147 Germany

**Keywords:** MARS500, Gut microbiota, Temporal dynamics, Life sharing, Confined environment, Space flight, Resilience

## Abstract

**Background:**

The intestinal microbial communities and their temporal dynamics are gaining increasing interest due to the significant implications for human health. Recent studies have shown the dynamic behavior of the gut microbiota in free-living, healthy persons. To date, it is not known whether these dynamics are applicable during prolonged life sharing in a confined and controlled environment.

**Results:**

The MARS500 project, the longest ground-based space simulation ever, provided us with a unique opportunity to trace the crew microbiota over 520 days of isolated confinement, such as that faced by astronauts in real long-term interplanetary space flights, and after returning to regular life, for a total of 2 years. According to our data, even under the strictly controlled conditions of an enclosed environment, the human gut microbiota is inherently dynamic, capable of shifting between different steady states, typically with rearrangements of autochthonous members. Notwithstanding a strong individuality in the overall gut microbiota trajectory, some key microbial components showed conserved temporal dynamics, with potential implications for the maintenance of a health-promoting, mutualistic microbiota configuration.

**Conclusions:**

Sharing life in a confined habitat does not affect the resilience of the individual gut microbial ecosystem, even in the long term. However, the temporal dynamics of certain microbiota components should be monitored when programming future mission simulations and real space flights, to prevent breakdowns in the metabolic and immunological homeostasis of the crewmembers.

**Electronic supplementary material:**

The online version of this article (doi:10.1186/s40168-017-0256-8) contains supplementary material, which is available to authorized users.

## Background

Human gut-associated microbial communities are necessary for several aspects of our physiology. A mutualistic configuration of the microbial ecosystem has a key role in metabolic homeostasis and in regulating the immune system, thus contributing strongly to shaping our health [1, 2]. In particular, the microbial-derived short-chain fatty acids (SCFA; mainly acetate, propionate, and butyrate) can act both locally and systemically, serving as energy substrates or signaling molecules, affecting satiety, energy production, and storage, and exerting a number of anti-inflammatory effects [3]. In this light, there is growing and highly topical interest in understanding the multiple factors, endogenous and environmental, that influence the composition and activity of the intestinal microbiota, with the intent to keep a health-promoting microbial arrangement along the course of our life [4]. However, our current knowledge of how microbial communities change over time in relation to host actions and behaviors, in health and disease states, is still limited. Two previous milestone studies have investigated the normal temporal dynamics of the gut microbiota in healthy volunteers in their daily lives, showing highly personalized microbial communities to be generally stable for months but quickly and profoundly perturbed, in a strictly personalized way, according to specific human experiences [5, 6]. More recently, researchers have highlighted substantial interaction and exchange over time between microbial communities of co-habiting humans [7–9]. These results suggest the potential of sharing life to lead to convergent temporal dynamics of the gut microbial ecosystem, paving the way to the possibility of steering the microbiota trajectories, by means of rational modulation of environmental factors. Within this context, Bashan et al. [10] explored the human microbial dynamics from an ecological perspective. Further supporting the controllable nature of the microbiota and therefore the feasibility of general microbiome-based interventions, the authors demonstrated that subjects with different species assemblages share similar, and probably universal, ecological dynamics of their microbial communities. Taken together, these recent findings indicate that environmental drivers can overcome, at least in part, the individual gut microbiota specificity. However, in this intricate scenario, longitudinal studies aimed at exploring the temporal variability of the human microbiota during prolonged life sharing in a confined and controlled environment are totally missing. Such studies would allow maximizing the impact of environmental factors on the individuality of the gut microbial ecosystem, shedding light on the degree of resilience of the individual gut microbiota profile and its pliable nature. The MARS500 project, with the longest ground simulation of an interplanetary space flight, provided us with a unique opportunity to address this issue, allowing tracing microbiota changes in six volunteer astronauts isolated in sealed compartments, in conditions of a regulated environment with the supply of mostly tinned foods similar to those used in the International Space Station, over 520 days of a virtual, but realistic, mission to Mars [11]. Crewmembers were asked to sample their faeces before entering the isolation module, at different time intervals throughout the whole mission and after the study completion, when they got back to their regular lives. Faecal samples were characterized by next-generation sequencing of the 16S ribosomal RNA (rRNA) gene, and longitudinal data were used to reconstruct the longest temporal dynamics of the human gut microbiota in confined isolation. Besides shedding some light on the dynamic behavior of the intestinal microbial ecosystem under controlled confinement, our findings on microbial ecology changes experienced by MARS500 crewmembers should be considered in programming future isolation experiments or real space flights, to help preserve the physical and psychological health of spacefarers, thus ensuring the mission accomplishment, which is a critical issue in long-term manned interplanetary space flights.

## Results

### Stability of the individual gut microbiota profile in confined environment

The intestinal microbial communities of the six crewmembers of the MARS500 project (subject 5001 to 5006) were tracked over time during the 520 days of ground-based space simulation. Longitudinal faecal sampling also included collection of stools before entering the isolation facility, and a number of samples after the exit from the modules, up to 6 months later. A mean of 27 faecal samples per crewmember were collected. Each sample was characterized via next-generation sequencing of V3–V4 hypervariable region of the 16S rRNA gene, allowing for a total of 5,377,450 high-quality sequence reads (mean per subject, 33,820; range, 7759–91,366). Reads were clustered into 54,836 chimera- and singleton-filtered OTUs at 97% sequence similarity.

**Fig. 1:**
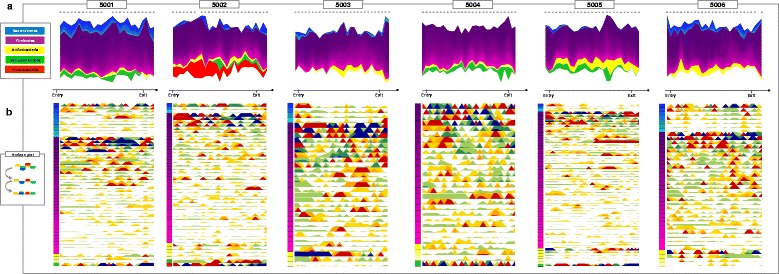
Gut microbiota dynamics in the crewmembers throughout the longest ground-based space simulation, MARS500. Graphical representation is based on Fig. 1 from David et al. [5]. **a** Stream plots showing OTU fractional abundances over time. Each stream is an OTU colored by phylum, whose width is proportional to the OTU relative abundance at a given time point (see *gray dots* above each plot and the timeline below with entry and exit flags). **b** Horizon graphs of the relative abundance variation of highly abundant OTUs over time. For each OTU, time series were median-centered and curves were divided into colored bands, whose width is the median absolute deviation, that were then overlaid, with negative values mirrored upwards. *Warm and cool colors* indicate relative abundance above or below the median, respectively, with *red* denoting greater abundance above the median than *yellow*, and *blue* greater abundance reduction below the median than *green. Squares* on the vertical axis are colored as in **a**. For the list of highly abundant OTUs, please see Additional file 2: Table S1

The gut microbiota dynamics were reconstructed across time and subjects, based on the variation of highly abundant OTUs, according to the normalization strategy described by David et al. [5]. As shown in Fig. 1, these trajectories revealed a strong individuality in the ecosystem structure and its dynamics during the prolonged isolated confinement in the MARS500 infrastructure, with a unique succession of individual microbial profiles. Even at phylum level, there were apparent microbial signatures that characterized each individual in his free-living conditions (i.e., before entering the isolation module) and accompanied him during and after the simulated interplanetary flight. The observed individual microbiota profiles at phylum level were consistent with the range of the phylum-level microbiota variation in the human population (Additional file 1: Figure S1). Specifically, Proteobacteria members were distinctive of subject 5002 (mean relative abundance across time series, 12.3%), Bacteroidetes were almost completely missing in subject 5004 (mean relative abundance across time series, 0.5%), and Verrucomicrobia were undetectable in the microbiota of subjects 5003 and 5006.

It should be pointed out that no detailed, individual-specific information on the micro- and macronutrient composition of the different variants of diet designed for the MARS500 astronauts throughout the whole project is available to the authors, making impossible any correlation with the microbial changes observed. However, based on available data, it is reasonable to assume that the diets were similar among astronauts across the mission and, thus, unlikely to make a major contribution to the inter-individual differences in the gut microbial dynamics.

Notwithstanding the strong individuality, the intestinal microbial communities of the six crewmembers shared 14 OTUs, all belonging to the Firmicutes phylum, and mainly to the *Lachnospiraceae* family (9 OTUs) (Additional file 2: Table S1; Additional file 3: Figure S2). All these OTUs were already present in the individual microbiota configurations prior to entry into the MARS500 module.

When exploring the variation of the beta (i.e., inter-astronaut) diversity of the gut microbiota over time, it is worthy to note that, unlike the weighted UniFrac distances that showed apparently random fluctuations, without a significant trend over time, the unweighted UniFrac values followed a downward trajectory, with a significant inverse association with the time spent in the MARS500 isolation facility (quantile median regression test: RC range, regression coefficients scaled to the full variation of UniFrac distances, −9362.98; RC sd, regression coefficients scaled to one standard deviation, 1900.01; *P* value generated by boot-strap analysis, 4E−5) (Additional file 4: Figure S3). In particular, immediately upon entry into the module, the median unweighted UniFrac distance was about 4% lower than the initial value (i.e., in free-living conditions) and reached a reduction of up to 9% after about 7 months spent in spacecraft.

### Temporal dynamics of gut microbiota components

The highly abundant OTUs used to reconstruct the individual microbiota trajectories over the MARS500 study were further analyzed by evaluating the OTU propensity to variation over time, as the magnitude of change above or below the median relative abundance (Fig. 1 and Additional file 2: Table S1).

Interestingly, some behavioral patterns of OTUs were shared among subjects. Specifically, OTUs attributable to *Roseburia faecis* (OTU_ID 84029, family *Lachnospiraceae*), *Faecalibacterium prausnitzii* (OTU_ID 543524, family *Ruminococcaeae*), and when present, *Akkermansia muciniphila* (OTU_ID 35867, family *Verrucomicrobiaceae*) were identified as highly variable during the whole period of confinement, regardless of the initial microbial configuration. In particular, the *F. prausnitzii* OTU was especially affected in the second half of the MARS500 mission, showing for all subjects the lowest relative abundance values around about 1 year of confinement. On the other hand, where detected, the *A. muciniphila* OTU decreased dramatically in the gut microbiota of all crewmembers when returning to their own free-living conditions after the end of the mission. The OTUs associated with *Bacteroides* (including *B. caccae*, *B. eggerthii*, *B. fragilis*, *B. ovatus*, *B. uniformis*, and unclassified species) were instead among the most perturbed in the initial phase of the mission, typically reaching for each subject the highest relative abundance values within the first 30 days of confinement. Conversely, the OTUs classified as *Dorea* (when available, *D. formicigenerans*, OTU_ID 181619) and *Coprococcus catus* (OTU_ID 616283) were highly stable in the intestinal microbiota of all crewmembers.

### Identification of microbiota steady states and ecological succession in time

**Fig. 2:**
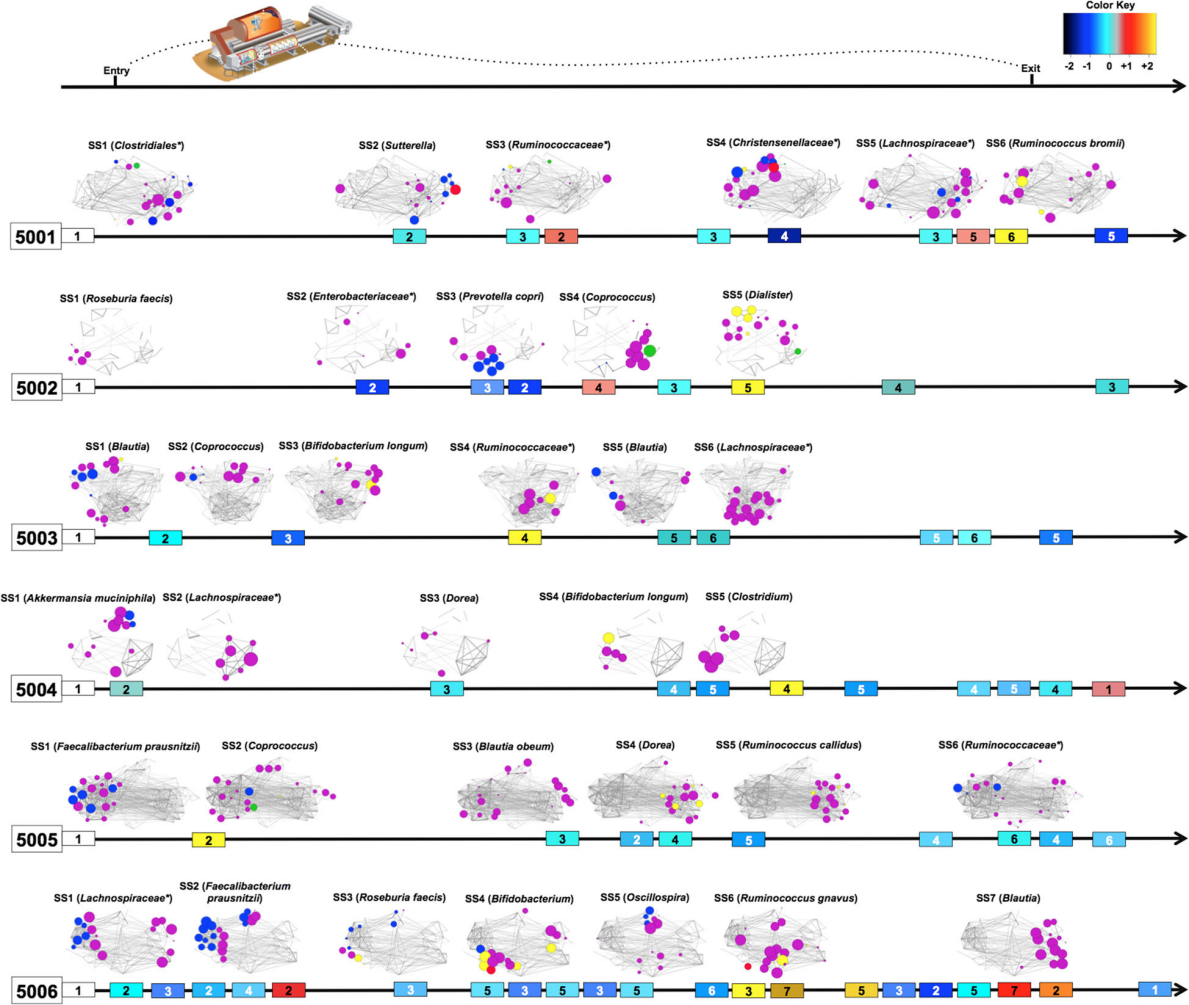
Ecological succession of microbiota steady states for the six MARS500 crewmembers over the entire mission simulation. For each crewmember, the temporal succession of the steady states, displayed as numbered boxes on the timeline (*black arrow*), is shown. Microbiota steady states were defined using a height threshold in individual hierarchical-Ward linkage trees, based on Jensen-Shannon distance. *Box color* is related to the dynamics of formation of the microbial structure of that steady state respect to the previous one, by evaluating the contribution of the individual microbial heritage and possible allochthonous microorganisms. Within each subject, for each steady state, except for the first one, the percentage of microbiota not deriving from the immediately preceding steady state was estimated using SourceTracker [34] and values were then normalized by a *z*-score approach. Higher values of z-score correspond to greater changes in the microbiota configuration with respect to the ordinary dynamics observed during the study (*yellow/red boxes*, see the color key on the top). Microbiota structures characterizing each steady state are displayed as Wiggum plot [13] of the most abundant OTUs, colored according to phylum membership as in Fig. 1a, grouped in co-abundance groups (CAO) (see Additional file 5: Figure S4). Steady state discriminatory OTUs were identified using Random Forests [12]; their taxonomy is shown above each Wiggum plot

Temporal dynamics of the gut microbiota were further investigated for each crewmember, by identifying microbial steady states and characterizing their ecological succession over time (Fig. 2 and Additional file 5: Figure S4). Steady states were interpreted in the context of recent theories of microbiome ecology, predicting that ecosystems may exist under multiple states (to be intended as “stable” configurations/equilibria of the microbial community) [5]. For each subject, steady states were identified through the same method described by David et al. [5], i.e., using a cluster-defining height threshold in a Jensen-Shannon Distance (JSD)-based tree. A total of 5 different steady states were identified for individuals 5002 and 5004; 6 for 5001, 5003, and 5005; and 7 for 5006. As already described for the individual microbial profiles, the phylogenetic structures of steady states were largely dominated by Firmicutes but retained a strong individual fingerprint in terms of taxonomic composition of the microbial communities, with variable percentages of Bacteroidetes, Proteobacteria, Actinobacteria, and Verrucomicrobia members (Additional file 5: Figure S4). According to our data, the temporal succession of steady states followed a highly personalized non-linear trajectory, with different temporal persistence and times of recurrence for each of them. Except for 5006, whose microbiota was deeply unstable, with steady states continuously alternating with one another, the intestinal microbial communities of the other crewmembers remained stable for periods lasting on average 2 months, with steady states generally recurring one up to three times over the entire sampling period. Interestingly, only for subjects 5004 and 5006, we observed a return to the initial microbiota configuration, after exiting the module, while new steady states were consolidated for the other crewmembers.

Steady state-based temporal dynamics of the crewmembers differed from each other also for the microbial ecology of the establishment of a steady state compared to the previous one. Specifically, we used a SourceTracker/*z*-score combining approach to measure the relative contribution of microbial sources other than indigenous ones in defining the steady state structures. According to our data, within each time series, the transition from one steady state to the next generally retained a strong individual signature, indicative of a rearrangement of the autochthonous microbial asset (contribution of the individual microbial communities up to 96%). Nevertheless, in each steady state-based trajectory, we could identify a major change event (three for subject 5006) in the microbial configuration, associated with a higher relative contribution of allochthonous microbial sources than indigenous ones (median contribution of the individual microbial communities, 10%). These major events were variously distributed along the individual timelines but occurred on average after 340 days (range, 330–360 days) of isolated confinement for subjects 5002, 5004, and 5006.

According to a Random Forests analysis [12], the vast majority of steady state discriminatory OTUs belonged to the *Lachnospiraceae* and *Ruminococcaceae* families (Fig. 2). The main exceptions were represented by *Bifidobacterium* OTUs that discriminated two steady states in the first half of the MARS500 simulation, for subjects 5003 and 5006, and one steady state of subject 5004 in the second half of the study and after the return to real life. On the other hand, Proteobacteria OTUs, specifically belonging to *Sutterella* and *Enterobacteriaceae*, discriminated steady states between 2 and 7 months of confinement for subjects 5001 and 5002, respectively.

To further explore the patterns of microbiota variation across the individual steady states over the MARS500 study period, for each crewmember, we established co-abundance associations of the most abundant OTUs and then clustered correlated OTUs into three or four co-abundance groups (CAO; Fig. 2 and Additional file 5: Figure S4) [13]. The differences in the succession of individual CAO profiles, with intra- and inter-CAO rearrangements, confirmed the personalized alternating of multiple reversible configurations of the gut microbiota in a single time series, mainly characterized by different proportions of *Ruminococcaceae* and *Lachnospiraceae* SCFA producers.

## Discussion

During long-term space flights, astronauts must share a confined and defined environment, where dealing with a number of organizational, technical, and psychophysiological issues, which can have negative implications for their health and the success of the whole mission. The MARS500 experiment, the longest high-fidelity space flight simulation ever conducted, with 520 days of confined isolation for a six-male multinational crew, represented an invaluable opportunity to investigate the human physiological adaptation to prolonged confinement while monitoring any problems spacefarers might face in real extended-duration space missions.

Within this context, in the present study, we explored the temporal dynamics of the gut microbiota in the six crewmembers participating to MARS500, across the entire duration of the mission, including the period before entering isolation modules, and after the return to regular life, for a total of at least 24 time points per subject and about 2 years of sampling. The MARS500 project allowed us to evaluate, for the first time, the impact of long-lasting isolated confinement conditions, with lack of social interaction, reduced contact with the environment, restricted resources, and mostly tinned food, ready or semi-ready for consumption, on the temporal variability of the intestinal microbiota. It should be mentioned that another study has recently attempted to characterize the faecal microbiota of MARS500 participants, but only seven time points and five subjects were taken into consideration [14], making it impossible to actually observe the ecological dynamics of the gut microbiota under strictly controlled conditions.

Our data demonstrate that the human gut microbiota is inherently dynamic, able to fluctuate between different phylogenetic configurations, even under the defined and controlled living conditions of an enclosed environment. As already observed in free-living subjects [5, 6], the temporal variation of the intestinal microbiota of crewmembers, inside the mock spacecraft, was found to be a highly personalized feature, with distinctive microbial assemblages and relative abundance profiles showing distinctive trajectories over time. Despite this strong inter-personal variability, all crewmembers shared, even before they entered the MARS500 module, about 20–40% of their highly abundant OTUs, and such percentage remained constant during life in the enclosed environment. On the other hand, according to the unweighted UniFrac beta diversity, the bacterial communities of the six astronauts became, to some extent, more similar to each other over time, suggesting a certain degree of convergence of the temporal dynamics of rare rather than abundant microbiota taxa in humans sharing a confined environment. Since the available information about the mission setup, especially with regard to air, module surfaces, and water supply system, leads to exclude the presence of factors that would favor sharing of microbes [15], we could extrapolate this sharing behavior to different confined environments other than the MARS500 modules.

To further investigate the gut microbiota dynamics in confined isolation, we identified individual steady states, which we define as subject-specific clusters of similar microbial configurations, and evaluated their ecological succession over time, providing a new interpretation of the temporal trajectories of the human gut microbiota. As described by David et al. [5], the initial microbiota state of an individual may indeed temporarily change, reverting to the original state after cessation of habitat perturbation, or persist in a new stable state, when the microbial communities are directly disrupted. The succession of these states is a peculiar and intrinsic feature of the individual, closely linked to the person’s life path. Analogously, according to our findings, in the confined environment of MARS500 modules, the steady state-based dynamics are unique for each subject, with a discrete number of steady states alternating in time with each other along non-linear and apparently stochastic routes. As already observed in free-living subjects [5], even under isolation conditions, steady states are formed mainly through relative abundance variations in autochthonous rather than allochthonous microbes, indicative of dynamic microbiota responses of “adaptive” rather than “innovative” nature [4]. Nevertheless, for each crewmember, it was possible to trace back a major change event in the microbial configuration, with a large inclusion of allochthonous microbial sources and not a mere rearrangement of indigenous ones. Interestingly, these events tended to occur in the second and third quarter of confinement, parallel to the major alterations already shown at the psychological, immune function, and intestinal health level [16–18].

Despite the overall personalized variation pattern of the gut microbiota of crewmembers over time, we observed temporal dynamics conserved among some strategic microbial components of individual ecosystems. Indeed, the overall increased relative abundance of *Bacteroides* species in all subjects in the very first stage of the mission, which had already been experienced by astronauts during the Skylab Medical Experiments Altitude Test (SMEAT) in a 56-day confinement study in 1975, and explained by the authors as a possible consequence of a stress situation is noteworthy [19]. Interestingly, early in the MARS500 mission, almost all crewmembers experienced one or more individual-specific disturbances of sleep quality, vigilance deficits, or alterations in sleep-wake timing and periodicity [20], suggesting a differential, but still stressful, context. *Bacteroides* is a major producer of propionate as well as phenolic acids, which are associated with benefits for human health [21]. Interestingly, this genus is often reported to be more represented in several stress conditions, with variations in luminal feeding, due to its eclectic capability of using mucus as glycan source. This capability allows *Bacteroides* spp. to persist even in upset ecosystems, supporting the resilience of the microbiota-host mutualism under conditions of decreased microbiota-accessible carbohydrates [22, 23]. On the other hand, well-known butyrate-producing members of the gut microbiota, such as *R. faecis* and *F. prausnitzii*, were found to continuously fluctuate in relative abundance in all crewmembers during the whole course of the mission, suggesting important variations in the pattern of SCFA production, with potential implications for the maintenance of the microbiota-host mutualistic relationship. Specifically, *F. prausnitzii* reached the lowest values around about 1 year of confinement, when psychological and biochemical data were consistent in highlighting particularly stressful circumstances, with the dominance of negative feelings, high levels of salivary cortisol, increased lymphocyte numbers and immune responses, and various degrees of positivity to the calprotectin test, used as a sensitive faecal marker of inflammation [16–18]. Even if no symptoms of intestinal inflammation were reported by crewmembers over the 520 days of mission simulation, the decrease of *F. prausnitzii* suggests an overall alert profile, with potential inflammation outbreaks, which in turn could lead to impairment of the host metabolic and immune homeostasis, and a high risk of onset of overt disease. However, it should be mentioned that OTUs specifically assigned to other important SCFA producers, including *Dorea* and *C. catus*, were basically unaffected, suggesting an alternative and lasting source of health-promoting microbial metabolites. Despite the aggravating psychological stress over time, the presence of these microorganisms may somehow compensate the above-described variations, ensuring a certain degree of SCFA provision and the maintenance of an immunomodulatory microbial profile. Further studies are needed to actually measure the faecal levels of SCFAs or, possibly, more comprehensive metabolomics studies should be performed during future mission simulations and real space flights, in order to promptly assess (and just as promptly correct) any unbalance in the repertoire of metabolites at the disposal of spacefarers, for the maintenance of their metabolic and immunological health. In the same way, shotgun metagenomics approaches should be applied, as they would enable greater resolution and sensitivity, leading presumably to more robust taxonomic assignments, even at species level, which is instead an inherent limitation of 16S rRNA profiling by the current amplicon sequencing technologies.

## Conclusions

Thanks to the unique opportunity to characterize the gut microbiota dynamics in the six astronauts of the longest ground-based space simulation project, MARS500, we can assert that the human intestinal microbiota retains a significant degree of temporal variability even under the strictly controlled conditions of an enclosed environment, oscillating between different configurations typically with rearrangements of autochthonous microorganisms. According to our findings, sharing life in a confined habitat does not compromise the individual specificity of the microbiota compositional layout, even in the long term, confirming the resilience of the individuality of the gut microbial ecosystem [24]. However, a combination of factors, including isolation and stress, force a conserved dynamic response of certain important components of the microbiota, with the potential to drive unbalances in the pattern of SCFA production, with cascading implications for the host metabolic and immunological homeostasis. Such alterations suggest a certain degree of playability of the gut microbiota structure and should be considered during future mission simulations and real space flights, with the purpose of keeping the microbiota-host mutualistic relationship.

## Methods

### MARS500 mission

The MARS500 programme was financed by the European Programme for Life and Physical Sciences in Space (ELIPS) and involves scientists from across Europe. It was performed by the State Scientific Center of the Russian Federation – Institute of Biomedical Problems (IBMP) of the Russian Academy of Sciences, and consisted of three isolation studies: a 14-day pilot study (completed in November 2007), a 105-day pilot study (completed in July 2009), and the main 520-day study, simulating a complete space flight to Mars, which is the focus of the present manuscript. A multinational crew, composed of six adult male volunteers (three selected by the Russian Federation, two by the European Space Agency, and one by the China National Space Administration; mean age, 31.8 years; range, 27–38 years), entered an isolation facility in the IBMP, Moscow, on June 3_,_ 2010, where they remained in continuous temporal and spatial confinement till November 4, 2011. During the stay in the spacecraft-like habitat, consisting of four hermetically sealed interconnected modules and one external module to simulate the Martian surface, they performed realistic activities of a roundtrip mission to Mars following a weekly work schedule, including, among others, operative work and meetings, exercise, scientific experiments covering the areas of physiology, psychology, biochemistry, immunology, biology, and microbiology, and even simulated emergency events. The parameters of the module habitat, determining physiologically comfortable proportion of the main components of gas media and their total pressure, complied with the standard “Cosmonauts’ habitat in a manned space ship”. Regularly (not less than once a month), the microbial contamination of the gas media was assessed, and selected surfaces from the habitable, medical, and utility module were swabbed over time, in collaboration with the crewmembers. The overall microbial load in the air and on different surfaces during the entire mission was found to be moderate compared to non-confined occupied rooms, sampled at the German Aerospace Center, private households and suburbs in Cologne (scientific experiment MICHAm, Microbial Ecology of Confined Habitats and Human Health, conducted in the context of MARS500) [15], and in any case, the CFU counts did not exceed the maximum allowed aboard the ISS [25]. Resources of water and food, whose composition reflected the diet used in the International Space Station (ISS) [26], were limited as in a real space flight. Specifically, two water supply systems were used: the system of drinking water supply (drinking water and for food cooking) and the system of domestic water for hygienic demands. The water quality in the first system was checked every 2 weeks. In the other system, water was from the centralized system of water supply of Moscow. No episodes of increased microbial contamination were reported. Regarding diet, the composition of food rations fulfilled the recommendations of World Health Organization (WHO) and also Russian-American norms on the food composition of rations for the crew of ISS. Specifically, three food rations were designed: (1) first variant, from the 1st till the 250th day of the mission (time of flight from Earth to Mars); (2) second variant, from the 251st till the 270th day for three crewmembers (simulation of the egress to the Martian surface); (3) third variant, from the 271st till the 520th day for the three crewmembers participating in the egress to the planet surface (returning to Earth), and from the 251st till the 520th day for the other three crewmembers. The food rations included different types of products, ready or semi-ready for consumption, by Russian, European, Korean, and Chinese firms, with up to four menu variants, providing on average 15.1% protein, 33.4% fat, and 51.2% carbohydrate. Detailed information about the entire project is available at http://www.esa.int/Our_Activities/Human_Spaceflight/Mars500 [11]. All scientific investigations carried out in the context of the MARS500 project were approved by the Ethics Committee of IBMP, and all crewmembers gave their written informed consent.

## Intestinal microbiota analysis

### Sampling

Crewmembers were asked to collect faecal samples 10 days before entering the isolation facility, at various time intervals throughout the entire 520-day simulation experiment, and after exiting the module up to 6 months later. Samples were collected in sterile vials, after cleaning the table top of the toilet with a hydrogen peroxide solution, washing hands with disinfectant gel, and wearing disposable gloves. In particular, for each time point inside the MARS500 modules, the vials were stored at 4 °C and, when samples from all six astronauts had been collected, they were moved outside the ground-based experimental facility (NEK facility) to the IBMP lab, where they were transferred to a −80 °C freezer into a Ziploc bag. Every 6 months, the samples collected were shipped to Italy in dry ice. For an overview of sampling times for each crewmember, please see Fig. 1.

### Microbial DNA extraction and Illumina MiSeq sequencing

Total bacterial DNA was extracted from faeces using the repeated bead-beating plus column method [27] with only minor modifications [28]. Briefly, cell lysis was achieved by introducing three 1-min steps in a FastPrep instrument (MP Biomedicals, Irvine, CA) at 5.5 movements per second, in the presence of 500 mM NaCl, 50 mM Tris-HCl pH 8, 50 mM EDTA, 4% (*w*/*v*) SDS, four 3-mm glass beads, and 0.5 g of 0.1-mm zirconia beads (BioSpec Products, Bartlesville, OK). After incubation at 95 °C for 15 min and centrifugation at full speed for 5 min to pellet stool particles, nucleic acids were precipitated by adding 10 M ammonium acetate and one volume of isopropanol. Seventy percent ethanol-washed pellets were resuspended in TE buffer, treated with 10 mg/ml DNase-free RNase at 37 °C for 15 min, and then subjected to protein removal and column-based DNA purification following the manufacturer’s instructions (QIAamp DNA Stool Mini Kit; QIAGEN, Hilden, Germany). The V3–V4 hypervariable region of the 16S rRNA gene was amplified using the 341F and 805R primers with added Illumina adapter overhang sequences as previously reported [29]. Amplicons were purified with a magnetic bead-based cleanup system (Agencourt AMPure XP; Beckman Coulter, Brea, CA). Indexed libraries were prepared by limited-cycle PCR using Nextera technology and further cleaned up as described above. The final library, prepared by pooling samples at equimolar concentrations, was denatured with 0.2 N NaOH and diluted to 6 pM with a 20% PhiX control. Sequencing was performed on Illumina MiSeq platform using a 2 × 300 bp paired end protocol, according to the manufacturer’s instructions. Sequencing reads were deposited as entire raw data in the National Center for Biotechnology Information Sequence Read Archive (NCBI SRA; BioProject ID PRJNA358005) and separately for each sample, along with available metadata, in the MG-RAST database (http://metagenomics.anl.gov/mgmain.html?mgpage=project&project=mgp79314).

### Computational and statistical analyses

Raw sequences were processed using a pipeline combining PANDAseq [30] and QIIME [31]. High-quality reads were clustered into OTUs at 97% sequence similarity using UCLUST [32]. Taxonomy was assigned using the RDP classifier against the Greengenes database (May 2013 release). The filtering of chimeric OTUs was performed by using ChimeraSlayer [33]. All singleton OTUs were discarded.

For the analysis of the gut microbiota dynamics over the entire Mars mission simulation, the normalization technique developed by David et al. [5] was used. Briefly, for each crewmember: (i) time points were normalized in the standard manner so that the sum of all fractional OTU abundances at a given time point was 1; (ii) highly abundant OTUs, accounting for 90% of median time point reads, were selected; (iii) each time point was normalized to a reference community that was computed for each sample based on other time points with a similar community structure. Specifically, reference OTU values were computed using a weighted median across time series, with time point weights set to be (1 − *j*)^2^ and *j* being the pairwise Jensen-Shannon Distance (JSD) score to the sample being normalized.

For each time series, steady states (i.e., “stable” configurations/equilibria of the gut microbial community) were identified clustering samples in a hierarchical-Ward linkage tree based on JSD and choosing a common tree height of 4 as the lowest across time series, that still allowed identifying sample groups (i.e., steady states) that (i) showed significant correlations between samples within the group (multiple testing using the q-value method) and (ii) were statistically significantly different from each other (permutational MANOVA using the JSD matrix as input, function Adonis of the vegan package in R). We assumed that such clusters approximated steady states of each individual microbiota, as microbial configurations with precise values of taxon relative abundance and defined functional roles. All steady states displayed significantly different inter-relationships from each other (*P* < 0.001). The taxonomic profiles for each steady state were generated taking the median value of relative abundance for each OTU in the samples being part of that steady state.

Within each subject, the dynamics of formation of the microbiota steady states was assessed by estimating, for each steady state (except for the first one), the percentage of microbiota not deriving from the previous steady state using SourceTracker [34], and then normalizing values by a z-score approach. In brief, for each crewmember, the relative abundance profiles of each steady state were alternately treated as sinks with the microbiota configurations of the immediately preceding steady state in the timeline of that subject as well as of the other individuals as sources. We then considered exogenous (i.e., other subjects and unknown) sources, obtaining a list of scores ranging from 0 to 1, and applied a *z*-score approach to these values, for each subject separately. In this way, when the *z*-score was 0, the change in the microbiota structure of that steady state corresponded to that observed on average; when the *z*-score was positive, the change was greater than observed on average; when the *z*-score was negative, the change was less than observed on average.

Co-abundance groups of OTUs (CAO) were determined as described in Claesson et al. [13], using OTUs with a mean relative abundance among steady state profiles more than 0.1%. Wiggum plots were created using Cytoscape 3.2.1. Steady state discriminatory OTUs were identified using the Random Forest machine learning algorithm [12]. Briefly, Random Forests is a powerful classifier that identifies the best subset of features (here, relative OTU abundance) at discriminating between categories (steady states within each subject).

## Additional files

Additional file 1: Figure S1. Comparison of the phylum-level gut microbial communities of the six MARS500 crewmembers in their free-living conditions with microbiota datasets from people from around the world. The relative abundance values of the five major phyla for the six astronauts immediately before entering the isolation module, were compared with publicly available data from the following previous studies: i) Schnorr et al. [35] (27 Hadza hunter-gatherers and 16 urban living Italian adults); ii) Yatsunenko et al. [36] (Malawian, Amerindian and US adults for a total of 185 individuals); iii) Martinez et al. [37] (40 adults from Papua New Guinea and 22 Western controls); iv) Gomez et al. [38] (28 BaAka hunter-gatherers and 29 Bantu agriculturalists from the Central African Republic); v) Obregon-Tito et al. [39] (Matses hunter-gatherers, Tunapuco agriculturalists and US adults for a total of 79 individuals); vi) Sankaranarayanan et al. [40] (38 American Indians and 20 non-native individuals); vii) Zhang et al. [41] (rural and urban adults from 7 ethnic groups throughout China for a total of 314 individuals). The MARS500 subjects are identified with colored dots, as in Additional file 5: Figure S4. The central dashed line represents the median, the lower and upper solid lines are the 5^th^ and 95^th^ percentile, respectively. (TIF 133 kb)

Additional file 2: Table S1. List of highly abundant OTUs in the gut microbiota of the six MARS500 crewmembers. For each OTU, ID and taxonomy are provided. Shared OTUs across subjects are shown in bold. (XLSX 17 kb)

Additional file 3: Figure S2. Venn diagram showing the distribution of shared OTUs among the intestinal microbial ecosystems of the six MARS500 crewmembers. For each astronaut, only highly abundant OTUs, accounting for 90% of median time points reads [5], were selected. For OTU ID and taxonomy, please see Additional file 2: Table S1. The Venn diagram was constructed using InteractiVenn tool [42]. (PNG 155 kb)

Additional file 4: Figure S3. Beta diversity of the gut microbiota of the six MARS500 crewmembers over the entire mission simulation. Box plots showing the distribution of inter-astronaut unweighted (A) and weighted (B) UniFrac distances before entering the isolation facility, during the 520 days of ground-based space simulation, and after exiting the modules, up to 6 months later. Only unweighted UniFrac distance values showed a significant inverse association with the time spent in the MARS500 isolation facility (quantile median regression test: RC range, regression coefficients scaled to the full variation of UniFrac distances, −9362.98; RC sd, regression coefficients scaled to one standard deviation, 1900.01; P value generated by boot-strap analysis, 4E-5). (TIF 241 kb)

Additional file 5: Figure S4. Assignment of co-abundance groups of the most abundant OTUs (CAO) and their evolution across the individual microbiota steady states. For each crewmember, CAO were determined by heat plot showing Kendall correlations between the most abundant OTUs clustered by Spearman correlation and Ward linkage, as described in Claesson et al. [13]. All CAO displayed significantly different inter-relationships from each other (*P* < 0.001, permutational MANOVA). Network plots show correlations between the identified CAO. Each node represents an OTU and its dimension is proportional to the relative OTU abundance (top plot) or the over-abundance relative to background (bottom plots for each microbiota steady state). Connections between nodes indicate positive and significant Kendall correlations between OTUs (*P* < 0.05). Line thickness is proportional to correlation strength. OTUs were filtered for those with >0.1% of mean relative abundance among the individual steady state profiles. (PDF 2955 kb)

## Abbreviations

CAO: Co-abundance group of OTUs
JSD: Jensen-Shannon Distance
OTU: Operational taxonomic unit
SCFA: Short-chain fatty acid
