# Supplementary figures and images for: Temporal dynamics of the gut microbiota in people sharing a confined environment, a 520-day ground-based space simulation, MARS500
Source: Microbiome. 2017 Mar 24;5:39. doi: 10.1186/s40168-017-0256-8 (PMC5366131; doi:10.1186/s40168-017-0256-8)

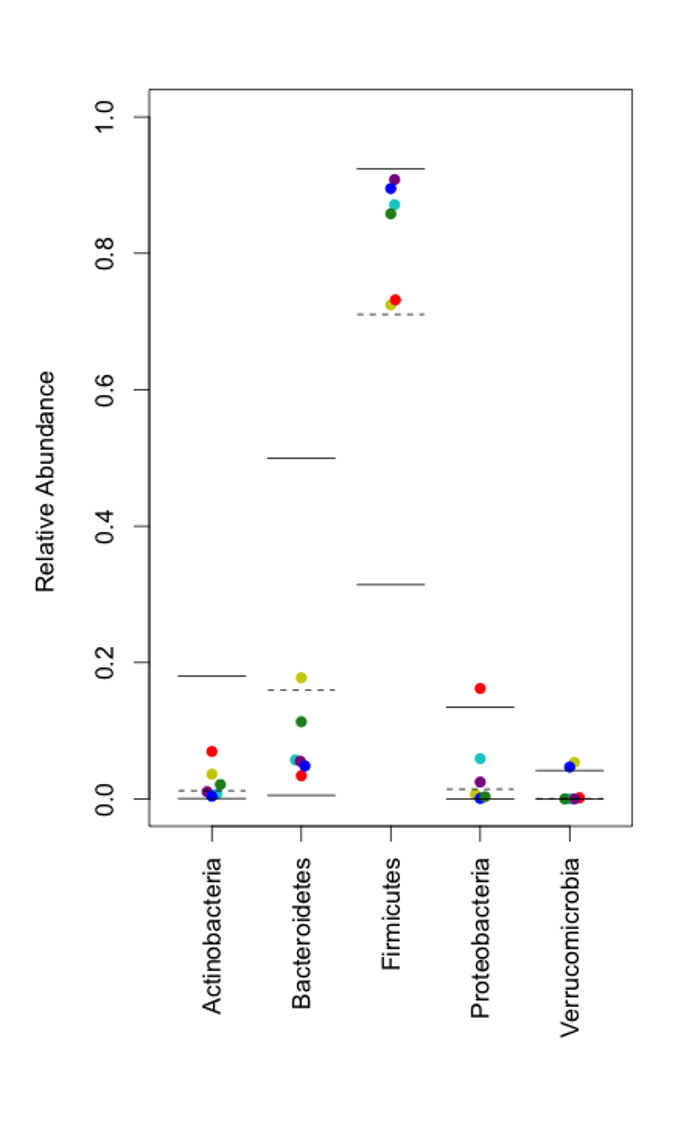

Supplement: Supplementary file 1 — Comparison of the phylum-level gut microbial communities of the six MARS500 crewmembers in their free-living conditions with microbiota datasets from people from around the world. The relative abundance values of the five major phyla for the six astronauts immediately before entering the isolation module, were compared with publicly available data from the following previous studies: i) Schnorr et al. [35] (27 Hadza hunter-gatherers and 16 urban living Italian adults); ii) Yatsunenko et al. [36] (Malawian, Amerindian and US adults for a total of 185 individuals); iii) Martinez et al. [37] (40 adults from Papua New Guinea and 22 Western controls); iv) Gomez et al. [38] (28 BaAka hunter-gatherers and 29 Bantu agriculturalists from the Central African Republic); v) Obregon-Tito et al. [39] (Matses hunter-gatherers, Tunapuco agriculturalists and US adults for a total of 79 individuals); vi) Sankaranarayanan et al. [40] (38 American Indians and 20 non-native individuals); vii) Zhang et al. [41] (rural and urban adults from 7 ethnic groups throughout China for a total of 314 individuals). The MARS500 subjects are identified with colored dots, as in Additional file 5: Figure S4. The central dashed line represents the median, the lower and upper solid lines are the 5th and 95th percentile, respectively. (TIF 133 kb) [file 40168_2017_256_MOESM1_ESM.tif]

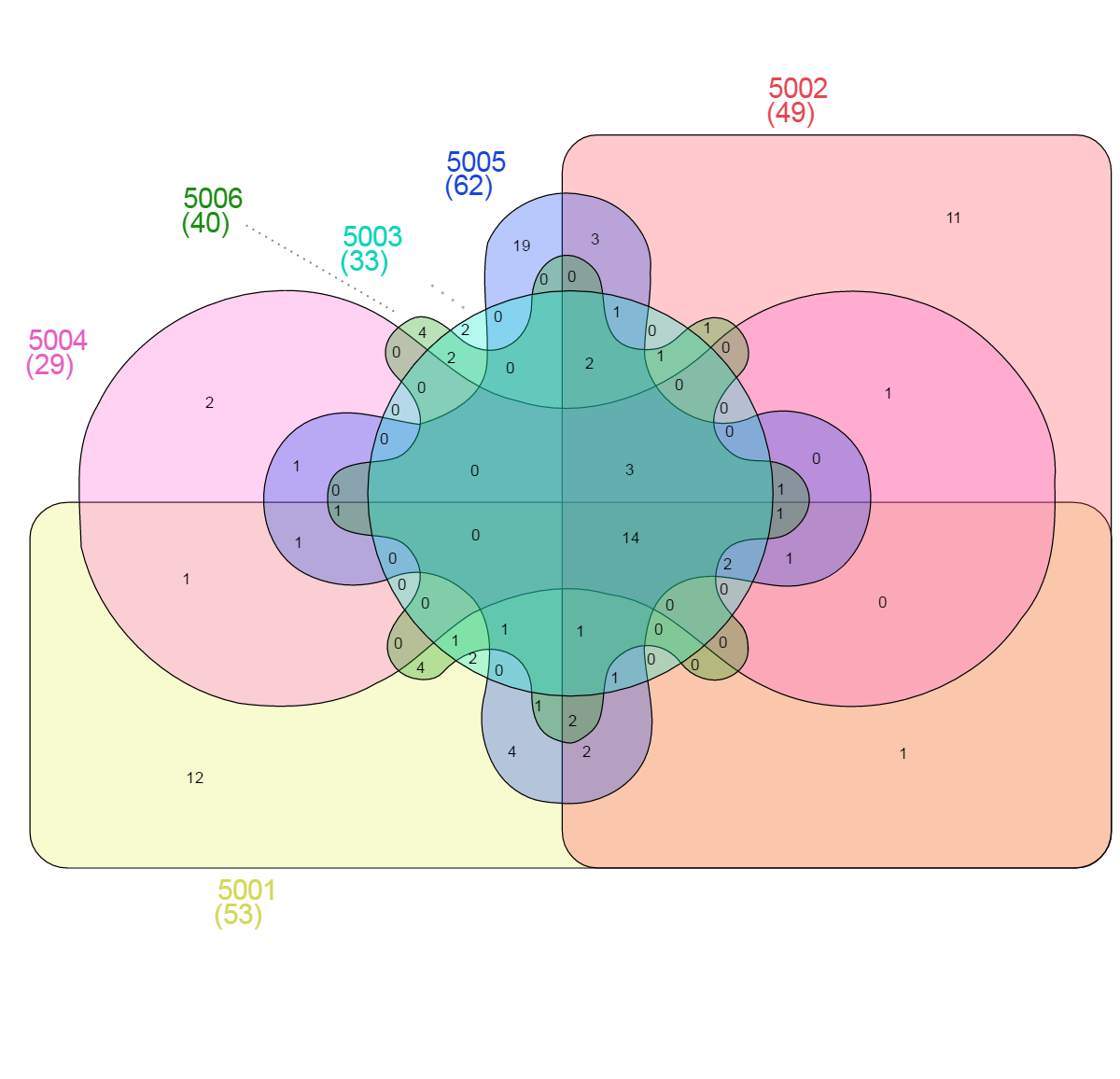

Supplement: Supplementary file 3 — Venn diagram showing the distribution of shared OTUs among the intestinal microbial ecosystems of the six MARS500 crewmembers. For each astronaut, only highly abundant OTUs, accounting for 90% of median time points reads [5], were selected. For OTU ID and taxonomy, please see Additional file 2: Table S1. The Venn diagram was constructed using InteractiVenn tool [42]. (PNG 155 kb) [file 40168_2017_256_MOESM3_ESM.png]

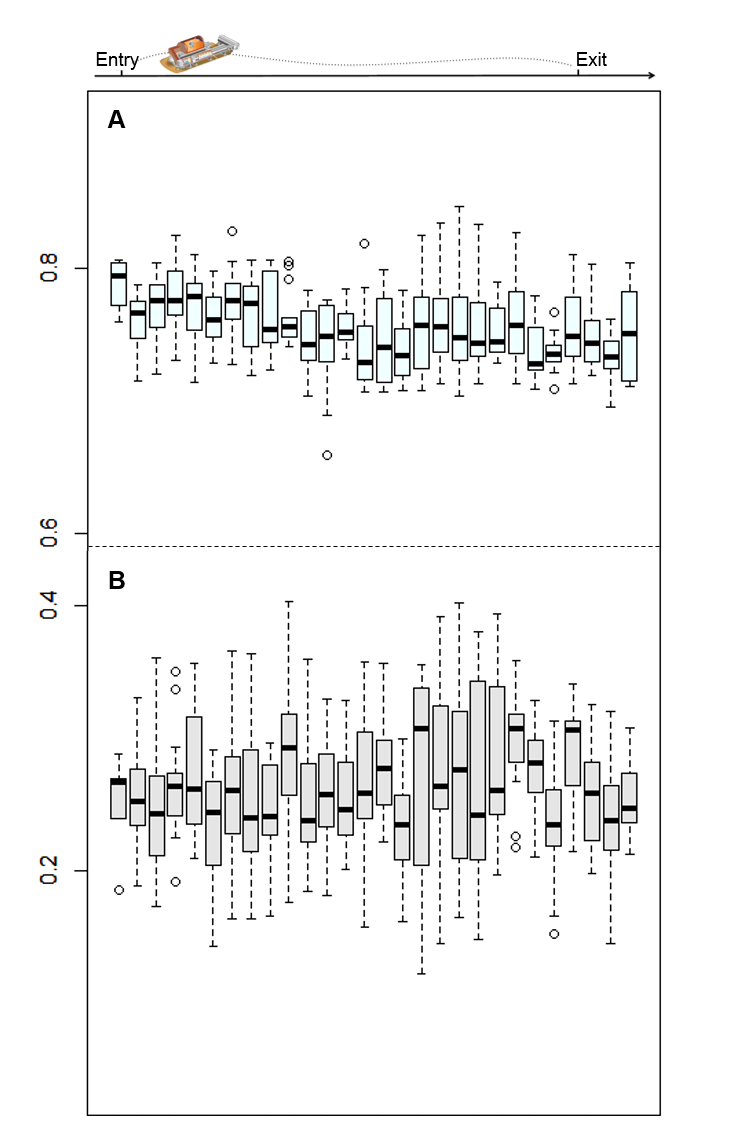

Supplement: Supplementary file 4 — Beta diversity of the gut microbiota of the six MARS500 crewmembers over the entire mission simulation. Box plots showing the distribution of inter-astronaut unweighted (A) and weighted (B) UniFrac distances before entering the isolation facility, during the 520 days of ground-based space simulation, and after exiting the modules, up to 6 months later. Only unweighted UniFrac distance values showed a significant inverse association with the time spent in the MARS500 isolation facility (quantile median regression test: RC range, regression coefficients scaled to the full variation of UniFrac distances, −9362.98; RC sd, regression coefficients scaled to one standard deviation, 1900.01; P value generated by boot-strap analysis, 4E-5). (TIF 241 kb) [file 40168_2017_256_MOESM4_ESM.tif]

Subject 1

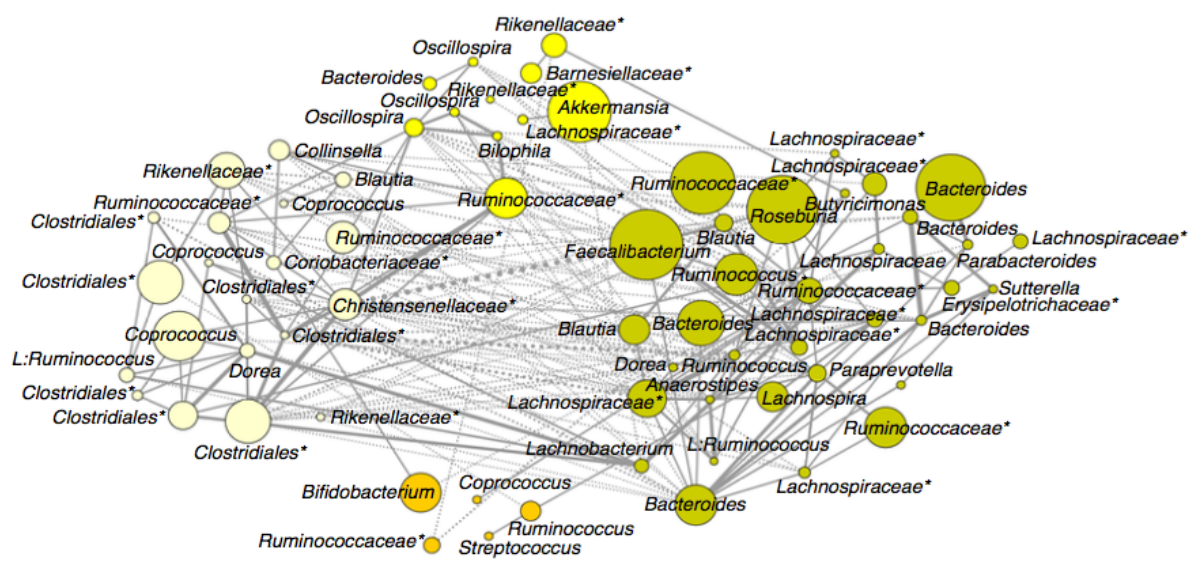

SS1

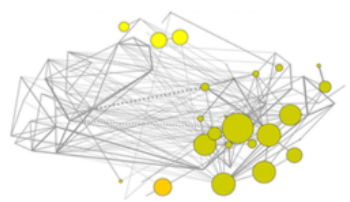

SS4

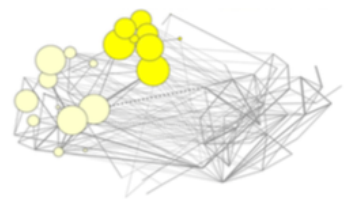

SS2

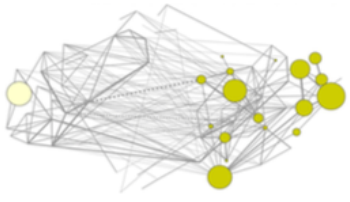

SS5

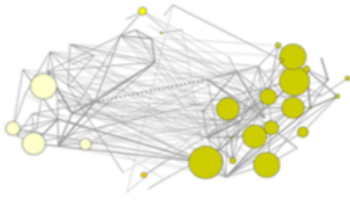

SS3

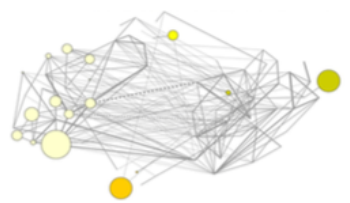

SS6

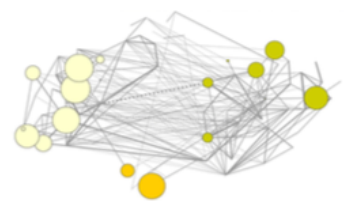

## Subject 2

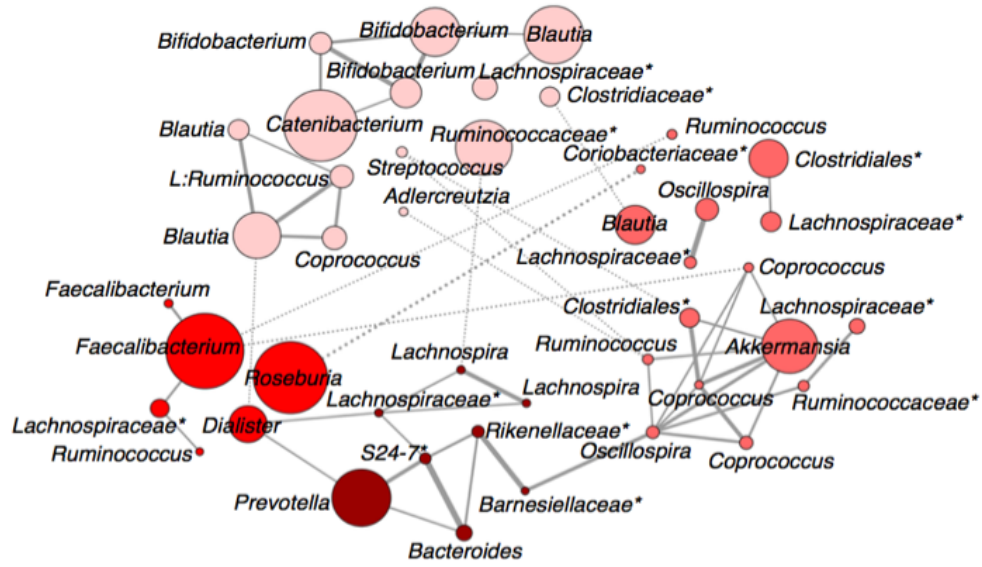

SS1

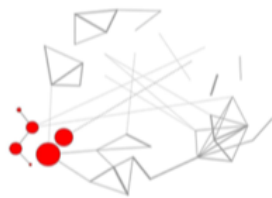

SS4

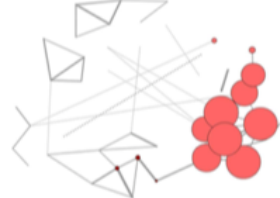

SS2

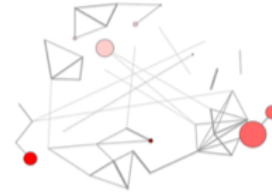

SS5

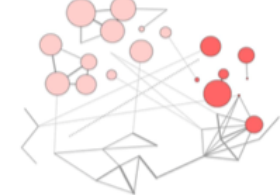

SS3

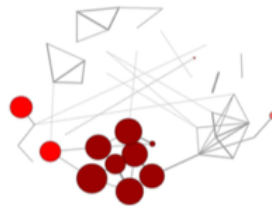

## Subject 3

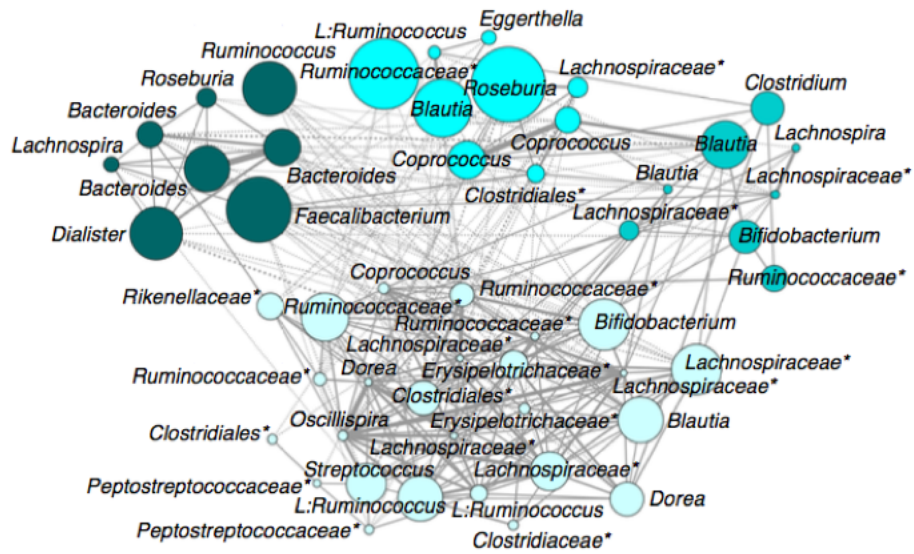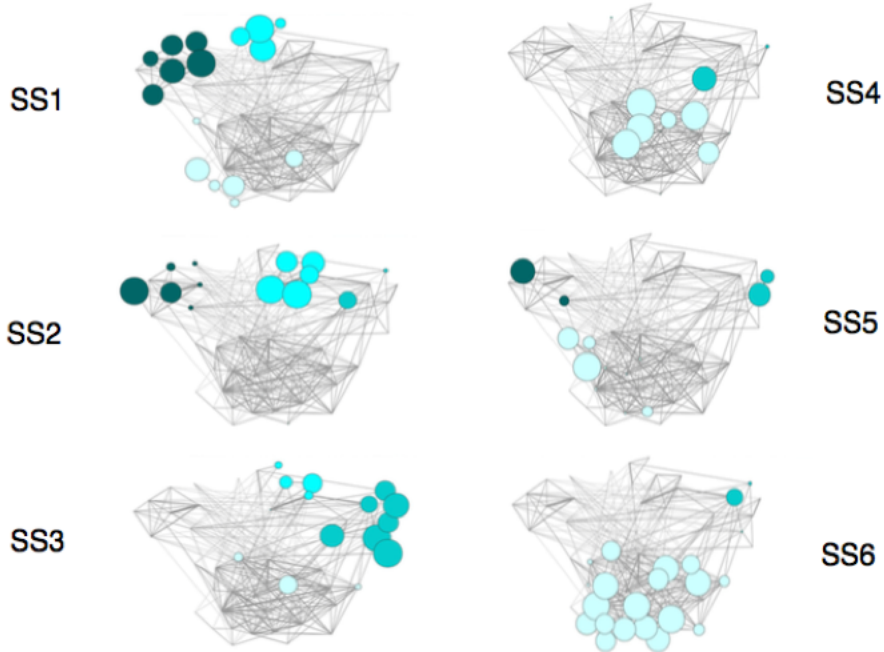

## Subject 4

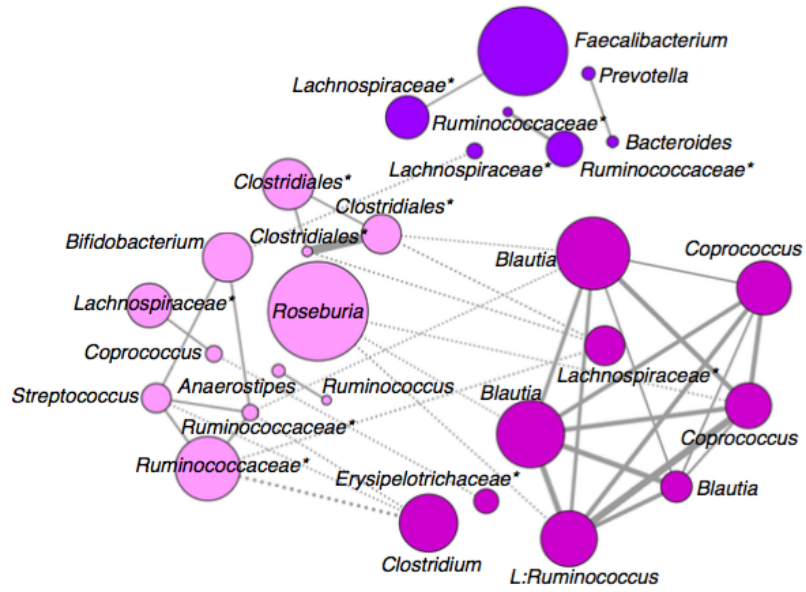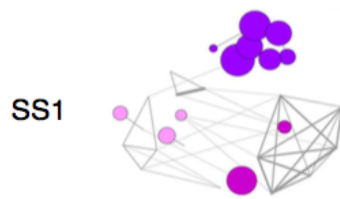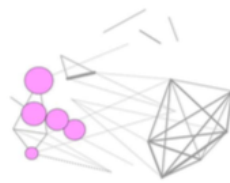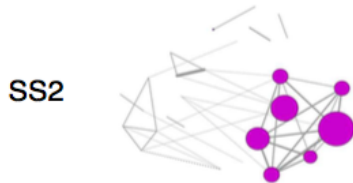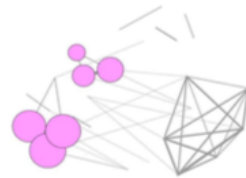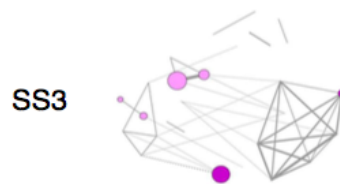

## Subject 5

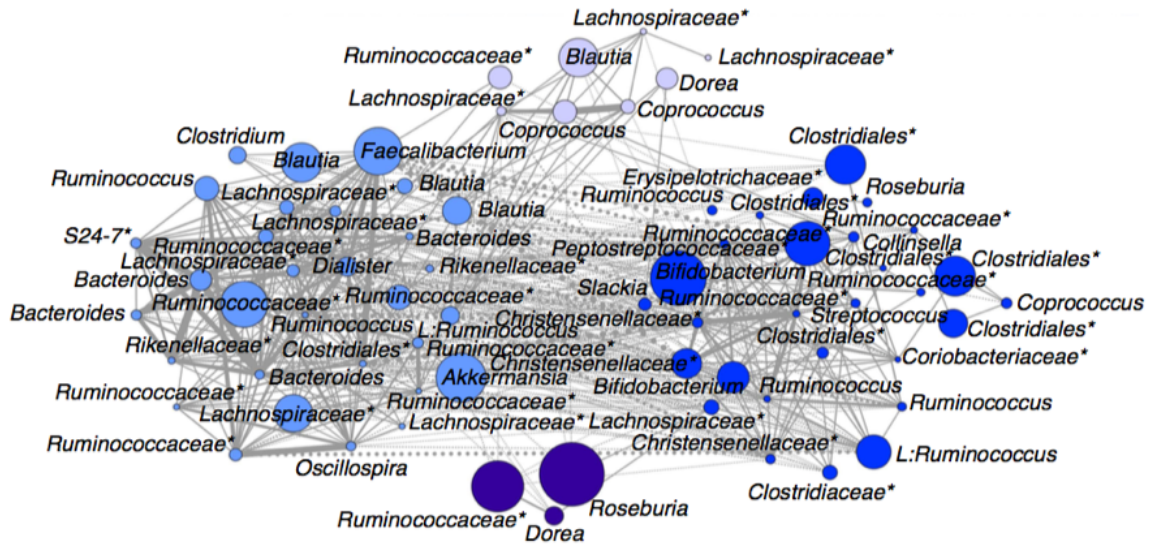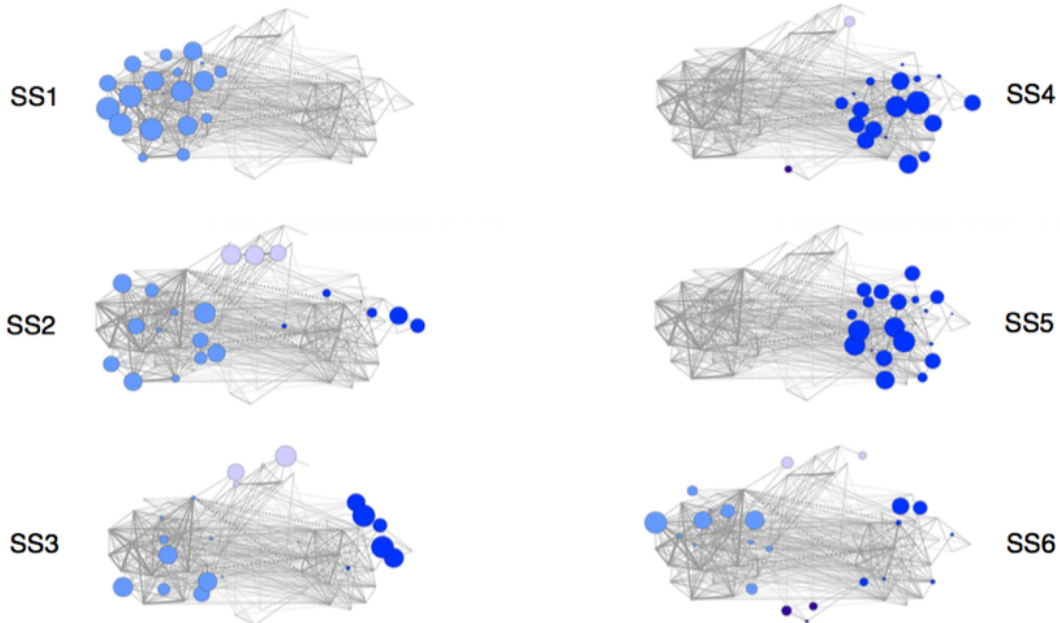

## Subject 6

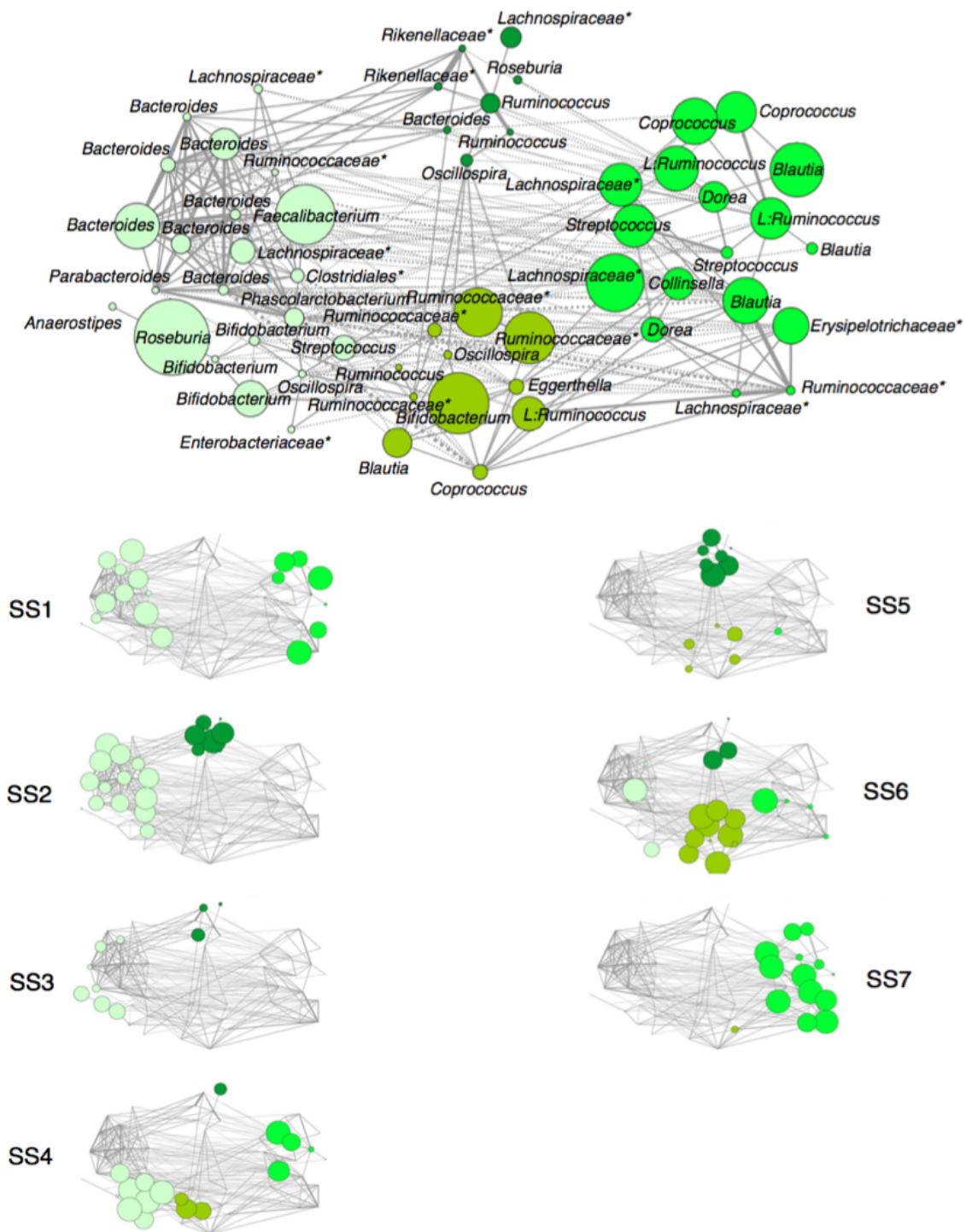

Supplement: Supplementary file 5 — Assignment of co-abundance groups of the most abundant OTUs (CAO) and their evolution across the individual microbiota steady states. For each crewmember, CAO were determined by heat plot showing Kendall correlations between the most abundant OTUs clustered by Spearman correlation and Ward linkage, as described in Claesson et al. [13]. All CAO displayed significantly different inter-relationships from each other (P < 0.001, permutational MANOVA). Network plots show correlations between the identified CAO. Each node represents an OTU and its dimension is proportional to the relative OTU abundance (top plot) or the over-abundance relative to background (bottom plots for each microbiota steady state). Connections between nodes indicate positive and significant Kendall correlations between OTUs (P < 0.05). Line thickness is proportional to correlation strength. OTUs were filtered for those with >0.1% of mean relative abundance among the individual steady state profiles. (PDF 2955 kb) [file 40168_2017_256_MOESM5_ESM.pdf]
